# Nanoparticle-Mediated Therapeutic Application for Modulation of Lysosomal Ion Channels and Functions

**DOI:** 10.3390/pharmaceutics12030217

**Published:** 2020-03-02

**Authors:** Dongun Lee, Jeong Hee Hong

**Affiliations:** Department of Physiology, Lee Gil Ya Cancer and Diabetes Institute, College of Medicine, Gachon University, Incheon 21999, Korea; sppotato1@gmail.com

**Keywords:** nanoparticles, nanomaterials, lysosome, ion channels, nanodrugs

## Abstract

Applications of nanoparticles in various fields have been addressed. Nanomaterials serve as carriers for transporting conventional drugs or proteins through lysosomes to various cellular targets. The basic function of lysosomes is to trigger degradation of proteins and lipids. Understanding of lysosomal functions is essential for enhancing the efficacy of nanoparticles-mediated therapy and reducing the malfunctions of cellular metabolism. The lysosomal function is modulated by the movement of ions through various ion channels. Thus, in this review, we have focused on the recruited ion channels for lysosomal function, to understand the lysosomal modulation through the nanoparticles and its applications. In the future, lysosomal channels-based targets will expand the therapeutic application of nanoparticles-associated drugs.

## 1. Lysosomal Target of Nanoparticles (NPs) and Modulation of NPs for Lysosomal Function

### 1.1. pH Alteration

The primary function of the lysosome is the degradation of proteins and lipids [1,2]. The regulation of lysosomal pH has been linked to various cellular functions including the degradation of intracellular compartments. For its cellular functions, lysosomal lumen has to be maintained at an acidic pH [3]. Degradation of proteins, which is a crucial function of the lysosome, is carried out by more than 60 kinds of lysosomal hydrolases [4], and these hydrolases are optimized for the highly acidic environment of lysosomes (between pH 4.5 and 5.0) [4,5]. The lysosome as a cellular digestive system eliminates the garbage materials from autophagy and phagocytosis [6,7,8]. Thus, destabilization of lysosomal pH thorough alkalization leads to cellular toxicity and even causes lysosomal storage disease (LSD) [9,10,11]. The application of NPs can mediate various cellular functions by modulating lysosomal pH. Gold NPs (AuNPs) are known to reduce lysosomal activity by alkalization of the lysosomal lumen [11]. This reaction triggers oxidative stress, mitochondrial damage, and decreases cell migration/invasion [11]. In particular, 50-nm sized AuNPs induce autophagosomal accumulation of LC3 and block p62 degradation [12]. Silver NPs (AgNPs) also suppress autophagic responses by decreasing transcription factor EB (TFEB) protein expression, which is followed by lysosomal alkalization [13]. In addition, rare earth oxide NPs (REONPs)-mediated alkalization induces the activation of interleukin-1β IL-1β by an inflammasome [14].

### 1.2. Cell Viability

The lysosome consists of a typical single phospholipid bilayer to control important cellular functions [15,16]. The lysosomal membrane acts as the connector to contact other compartments such as autophagosome [17,18], mitochondria [19], and endoplasmic reticulum (ER) [20]. On the lysosomal membrane, numerous proteins play important roles such as the mammalian target of rapamycin complex 1 (mTORC1) (nutrient sensing) [21], V-ATPase (Vacuolar type of H^+^-ATPase) (pH homeostasis) [22], and ion channels/transporters [23]. In addition, deficiency of several lysosomal membrane proteins trigger various diseases such as the Danon disease (lysosome associated membrane proteins, LAMP-2) [24], malignant infantile osteopetrosis (the chloride channel 7, CLC-7) [25], and actin myoclonus-renal failure syndrome (lysosomal integral membrane protein-2) [26]. Damaged lysosome mediates lysosomal membrane permeabilization (LMP), which contributes to cell death [27,28] and induces several diseases such as LSD and other neurodegenerative disease [29,30,31]. Numerous NPs can have membrane damaging effects such as polystyrene NPs (PNPs) [32,33,34], silica dioxide (SiO_2_) NPs [35], titanium dioxide (TiO_2_) NPs [36], and Gd_2_O_3_:Eu^3+^ (Gd_2_O_3_) NPs [37], and, thus, cause cellular malfunctions. The PNPs (especially positive-charged) block autophagic flux [32], and release cathepsins (proteolytic enzymes), which induce cell death [34]. In addition, the LMP of other NPs reveal NACHT, LRR and PYD domains-containing protein 3 (NLRP3) inflammasome (SiO_2_NPs) [35] and necrosis (Gd_2_O_3_ NPs) [37].

### 1.3. Protein Activity and Expression

Various lysosomal functions are mediated by more than 200 integral lysosomal membrane proteins [4], including (1) the mechanistic target of mTORC1, which is activated by nutrient starvation [28,38], and acts as a negative regulator of autophagy [28,39], and (2) LAMPs, which protect the lysosomal membrane against lysosomal hydrolases not to degrade [40]. NPs induce an inhibitory effect on the mTORC1 pathway to activate autophagy: AgNPs (decreases lysosomal protease activities) [41], Zinc oxide (ZnO) NPs (induces macrophage cell death) [42], and REONPs (induces lysosomal imbalance by TFEB nucleus translocation) [43]. ZnO NPs induce an aberrant expression pattern and de-glycosylation of LAMP-2 by ZnO-induced reactive oxygen species (ROS), which trigger cell death in lung epithelial cells [44]. Additionally, NPs modulate lysosomal motility [45]. Lysosome movement reveals two directions: toward the peripheral cytoplasm (anterograde) [46,47] and juxtanuclear region (retrograde) [48]. To carry out autophagic flux, lysosomes have to move to the juxtanuclear region [22,38], and the dynein complex is the motor protein for retrograde transport [49]. Treatment with carbon nanotubes decreases the expression of synaptosomal-associated protein (SNAP), which is a regulating factor of dynein [50] that blocks retrograde transport and, thus, the autophagic pathway [45]. Taken together, the lysosomal pathways of NPs and occupied proteins may mediate numerous functions. Thus, careful and more extensive consideration of lysosomal-associated NPs needs to be done.

### 1.4. Accumulation of NPs

Toxic cellular components, such as cytoplasmic macromolecules, damaged or misfolded proteins, and other worn-out organelles, are removed by lysosomes to maintain metabolic homeostasis [3]. Thus, the degradation role of lysosomes is essential for carrying out cellular homeostasis [51] including lipid catabolism [52], cell growth [53], and neurotransmission [54]. However, several NPs interrupt lysosomal degradation and deposit the lysosomal compartment in the cytoplasm. Exposure to AgNPs and copper oxide (CuO) NPs can induce agglomeration of lysosomes and subsequent cellular damage, which leads to cell death in human lung alveolar epithelial cells [55] and human umbilical vein endothelial cells [56]. In addition, NPs can accumulate in lysosomes. SiO_2_NPs and PNPs impair cell viability and induce lysosomal swelling, which is followed by their accumulation in lysosomes and triggers lysosomal dysfunction and apoptosis [57,58].

## 2. Regulation of Lysosomal pH and Its Physiological Function

The lysosomal pH gradient is generated and maintained by movement of hydrogen ions (H^+^) into the lysosomes through the action of vacuolar-type ATPases (V-ATPases) [59], which is supplemented further by movement of other ions [5]. Thus, for effective and continuous movement of H^+^ into the lysosome, an accompanying counter-ion movement is necessary [5].

The lysosomal V-ATPases consists of two domains: V_1_ domain, which hydrolyses ATP, and the V_0_ domain, which translocates H^+^ ions across the lysosomal membrane [60]. The catalytic domain V_1,_ drives a rotary H^+^ transport motor by hydrolyzing ATP with translocation of H^+^ [61,62]. In this case, the V-ATPase rotor is operated in only one direction with an irreversible ATP hydrolysis due to the movement of H^+^ from cytosol to the lysosomal lumen [5]. The continuous V-ATPase-mediated H^+^ pumping generates a positive charge in the lysosomal lumen, which inhibits any further movement of H^+^ [63]. To dissipate this membrane potential, other ions have to be transferred in the opposite direction, and this process is referred to as the counterion flux [5,63]. Counter ion movement is suggested as both entering anions and exiting cations through the lysosomal lumen [5]. One important counter ionic candidate is chloride, transferred by CLC-7, as attenuation of CLC-7 leads to lysosomal dysfunction such as LSD and osteopetrosis [25,64]. Another candidate counter ion is K^+^, transferred by TMEM175. Its mutation induces neuronal degeneration and LSD [65]. The R740S mutant osteoclasts, mutated in the V-ATPase α3 subunit, possess a higher lysosomal pH, and shows altered mTORC expression (increase in basal protein level and decrease of gene expression) and activity, which, in turn, plays a key role in cell proliferation [57,66]. Additionally, acidification of lysosomes can induce macrophages to secrete *N*-acetyl-*β*-D-glucosaminidase through lysosomal exocytosis [67,68], which includes absorption of cytochrome c in rat kidney during renal metabolism [69], and transport of cystine, the product of protein degradation by cathepsin, from lysosomes to cytosol [70]. Thus, alteration of lysosomal pH can be like a commander’s order to modulate the cellular life cycle.

## 3. Lysosome-Associated Ion Channels for Lysosomal Function

**Figure 1:**
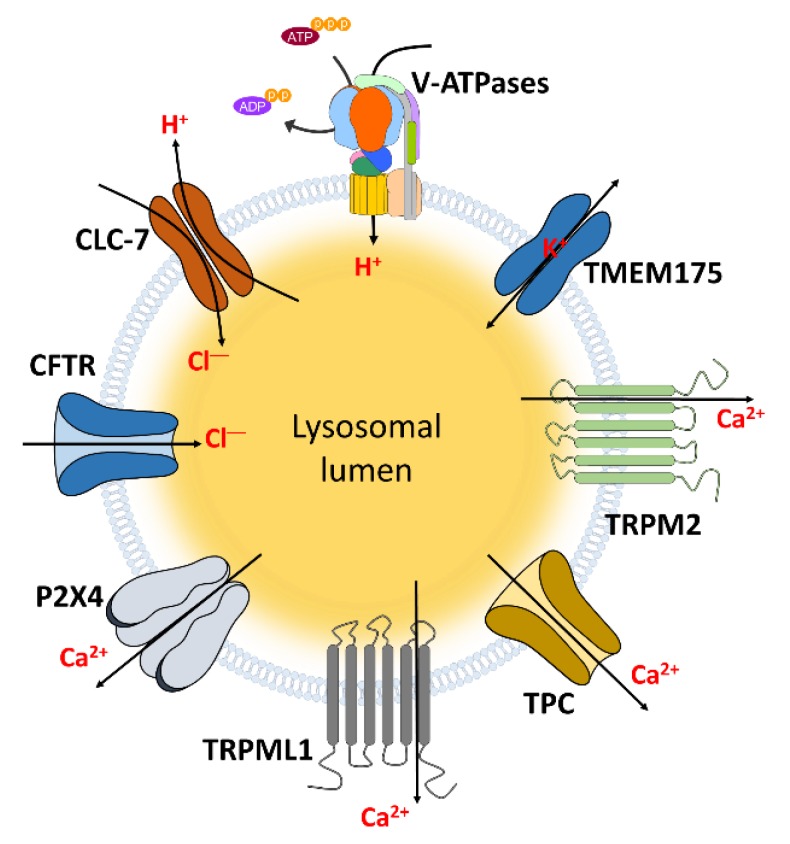
The channels localized in lysosomal membrane to transport ions. These channels and transporters can regulate lysosomal and cellular functions through transporting and maintaining hydrogen, chloride, Ca^2+^, and potassium which indicated in Table 1.

The lysosomal function is modulated by the ion movement and subsequent pH regulation. This movement is accomplished through various ion channels (Figure 1). We have previously reported application of NPs on various channels [71]. In this section, we summarize the recruited channels for lysosomal function to understand the lysosomal modulation through the NPs (Table 1).

### 3.1. CLC

CLC channels are the chloride channels that play a critical role in lysosomal function. CLC channels consist of two major isotypes: plasma membrane-associated (CLC-1, -2, and -Ka/-Kb) and intracellular organelle-associated (CLC-3 to CLC-7) [114,115]. Among the intracellular organelle-associated CLC, CLC-3 channel promotes lysosomal acidification and induces bone resorption [72,73]. Deletion of CLC-6 DNA leads to LSD in neuronal cells [74]. Particularly, CLC-7 channel—a chloride/H^+^ antiporter—is a well characterized CLC channel that serves as a major pathway for chloride ion, and in lysosomes [116,117,118]. As mentioned above, CLC-7 has a regulatory role in lysosomes and inhibition of CLC-7 leads to various diseases such as LSD and osteopetrosis [25,64,81,82,83,84]. Lysosomal acidification is essential for osteoclast-mediated bone resorption. Mutations in the CLC-7 channel can inhibit the lysosomal acidification in an osteoclast [75,76] and trigger osteopetrosis [81,84]. CLC-7-deficient mice show LSD and neurodegeneration, which is followed by retinal degeneration [64,82]. For lysosomal acidification, the CLC-7 channel has to be trafficked to the lysosomes, supported by Ostm1 [119]. Acidification of lysosomes and activation of microglial cells both require CLC-7 channel trafficking to lysosomes for the degradation of amyloid-β peptide (fAβ) deposition, which drives Alzheimer’s disease (AD) [79,80]. Additionally, it has also been reported that deletion of the CLC-7 channel reduces the dentinogenesis and dental bone formation [77,78].

### 3.2. Cystic Fibrosis (CF) Transmembrane Conductance Regulator (CFTR)

CFTR is an ATP-binding protein, which is regulated by its phosphorylation regulatory (R) domains, and transports chloride among other anions including bicarbonate ion (HCO_3_^−^) [120,121,122,123,124]. Mutation of CFTR causes defects in fluid secretion and is responsible for the genetic disease CF [120,124,125]. A prevalent cause of CF results from a deletion of the 508th positioned phenylalanine (ΔF508) even though several other mutations have been identified in CF [120,121,125]. CFTR has been reported to support lysosomal acidification and is localized in intra-organellar components, including ER, Golgi, and endo/lysosomes [126,127]. In CF cells, which have a ΔF508 mutation in CFTR, lysosomal pH is higher than in normal cells [85]. CFTR-null macrophages showed a defective killing function of internalized bacteria by inhibiting phago-lysosomal fusion [86]. Typically, these macrophages kill bacteria by phago-lysosomal ingestion, which is followed by lysosomal acidification [86,127]. This suggests that CFTR-mediated lysosomal acidification can regulate bacteria-killing activity of macrophages. Additionally, activation of CFTR leads to re-acidification of alkalinized lysosomes in retinal pigmented epithelial cells, which suggests it is a useful target for lysosomal clearance [128].

### 3.3. TRPs

The TRP channels, grouped into six subfamilies of TRPC, TRPV, TRPM, TRPA, TRPP, and TRPML (transient receptor potentials canonical, vanilloid, melastatin, ankyrin, polycystic, and mucolipin, respectively), are cation permeable channels, composed of six transmembrane domains [129,130]. These channels, with their numerous subtypes, have various functions. In particular, TRPM2 and TRPML1-3 play important roles in lysosomes (only four subtypes are localized in the lysosomal membrane) [104,129,131].

#### 3.3.1. TRPM2

**Figure 2:**
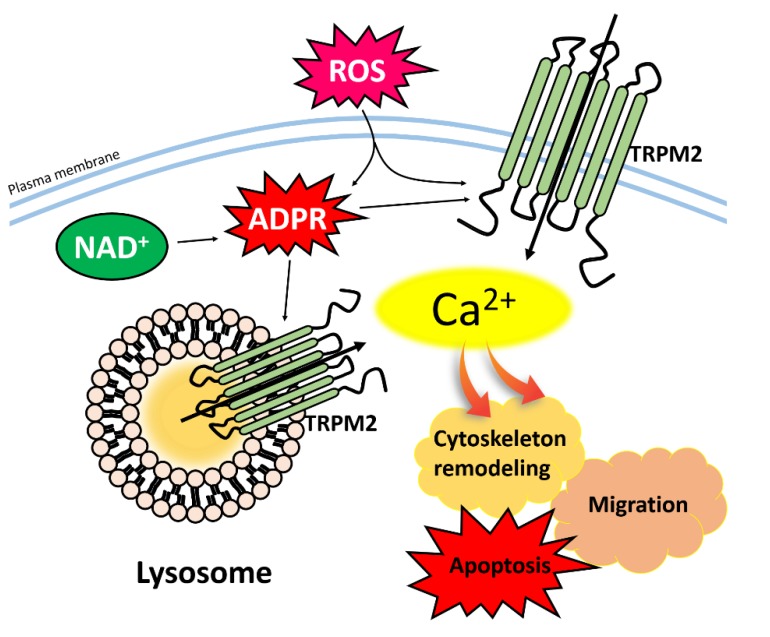
Activation of TRPM2 channel and cellular function. ADPR, NAD, and ROS induce up-regulation of intracellular Ca^2+^ concentration through the TRPM2 and, subsequently, mediate with cell migration, cytoskeleton remodeling, and apoptosis. Abbreviations: TRPM2: Transient receptor potentials melastatin 2; ADPR: Adenosine diphosphate ribose; NAD: Adenine dinucleotide; ROS: Reactive oxygen species.

The TRPM2 channel is one of the TRPM family cation channels, which is activated by adenosine diphosphate ribose (ADPR) [132,133,134,135], adenine dinucleotide (NAD) [132,136], ROS [135,136,137], and extra/intra-cellular Ca^2+^ [138,139,140]. TRPM2 is located to numerous tissues and cellular compartments and has various activation mechanisms (Figure 2). Thus, the Ca^2+^ ion influx through TRPM2 plays multifunctional roles [141,142,143]. TRPM2 is also localized on the lysosomal membrane and modulates cellular functions such as cell migration, cytoskeleton remodeling, and apoptosis [87,88,89]. On the lysosomal membrane of dendritic cells (DC), TRPM2 releases Ca^2+^ ions to the cytoplasm to mediate optimal DC maturation and DC migration and homing to lymph nodes [87]. H_2_O_2_-induced Ca^2+^ influx increases through lysosomal TRPM2 and triggers actin remodeling, which, subsequently, activates cell migration, even though the extracellular Ca^2+^ entry does not affect the cytoskeletal remodeling [88]. Additionally, lysosomal TRPM2 Ca^2+^ ion release in pancreatic β cells induces apoptosis [89]. On the other hand, plasma membrane-localized TRPM2 mediates lysosomal damage via LMP and is associated with NLRP3 inflammasome-activation and mitochondrial fission [90,91].

#### 3.3.2. TRPMLs

TRPMLs (all three subtypes, TRPML1-3) are the main cation channels in the endo-lysosomal membrane, and regulate endo-lysosomal cation homeostasis, trafficking, and other cellular functions including intracellular compartment-acidification [104,131,144,145,146,147,148,149,150,151]. At the same time, TRPML1 is the main channel for lysosomal Ca^2+^ ion releases. TRPML2 and TRPML3 also have important roles in endosomal vesicles: regulation of TRPML2 is involved in the Arf6 recycling pathway [152], innate immune response [153], and B cell development [144,154]. The regulation of TRPML3 is involved in sensing lysosome neutralization [155], hearing functions [156,157], membrane trafficking, and autophagy [158]. Lysosomal Ca^2+^ ion-release through TRPML1 plays a major role in autophagy, mediated by starvation-induced mTORC1 deactivation and TFEB-induced autophagic gene expression [159,160] with simultaneous regulation of lysosomal acidification [92].

TRPML1 can regulate various cellular functions such as large particle phagocytosis through lysosomes [96], autophagosome biogenesis [161], elimination of bacterial pathogens through lysosome activation [162,163], bone remodeling in osteoclastogenesis [94], gastric acid secretion [93], and coronary arterial myocytes apoptosis [95]. In addition, the TRPML1 can reduce the enlargement of the lysosome by activating calmodulin [164]. Since TRPML1 has numerous functions, its deficiency can trigger various diseases, including stomach hypertrophy and hypergastrinemia [93], LSD [97,98,99], mucolipidosis type IV [100,101,102], Niemann-Pick disease type C (NPC) [97], and AD [103].

### 3.4. TMEM175

Intra-organelle K^+^ channel TMEM175 was recently identified in endosomes and lysosomes and is involved in the modulation of luminal pH stability and autophagosomes [165]. Deficiency of TMEM175 results in dysregulated lysosomal pH, impaired autophagosome clearance, and mitochondrial dysfunction in the neuronal system [166]. In addition to TMEM175, Ca^2+^-activated large conductance K^+^ channel also localizes to the lysosome and is involved in lysosomal Ca^2+^ signaling and lipid accumulation [104,105], which suggests lysosomal K^+^ channels can be considered the new target of neurodegenerative diseases such as LSD.

### 3.5. Other Ca^2+^ Channels

#### 3.5.1. Two Pore Channels (TPCs)

TPCs are the key components of Ca^2+^ signaling in the endo-lysosomal system including TRPML and TRP channels and have been extensively reviewed in various studies [167,168,169]. The TPC1-3 are identified in the endo-lysosome [170,171,172,173,174] and stimulated by nicotinic acid adenine dinucleotide phosphate and phosphatidylinositol 3,5-bisphosphate [171,175,176,177,178]. The roles and pathways of TPCs have been addressed in various organs and biological systems. The inhibition of the TPC channel abolishes the migration of metastatic cancer cells by disrupting the trafficking mechanism of β1-integrin and the formation of leading edges [179]. The TPCs are involved in the autophagic flux of mouse cardiomyocytes [180]. It has been discussed that TPC2 is involved in autophagy progression, cancer cell migration, and cellular pigmentation [106,107,108]. Additionally, signaling events of Parkinson’s disease involve the regulation of TPCs in trafficking [109,110].

#### 3.5.2. P2X4

The P2X4 receptor is expressed ubiquitously in cells from immune, nervous, muscle, and vascular systems [181,182,183]. The P2X4 is stable within the acidic environment of the lysosome and also traffics to the plasma membrane to enhance the phagocytic function [181,184]. P2X4 is activated by ATP and inhibited by the luminal acidic pH in the lysosome [185]. P2X4 consist of an ATP-activated Ca^2+^ channel and is involved in calmodulin activation to promote endo-lysosomal fusion of intracellular organelles [111,112]. P2X4 is also involved in liver fibrogenesis [113] and alcohol-induced microglial damage [186]. Although P2X4 has been associated with ATP-dependent signaling in the endo-lysosome, further studies are still needed in the future.

## 4. NP-Induced Proton Sponge Effect through Ion Channels in the Tumor System

**Figure 3:**
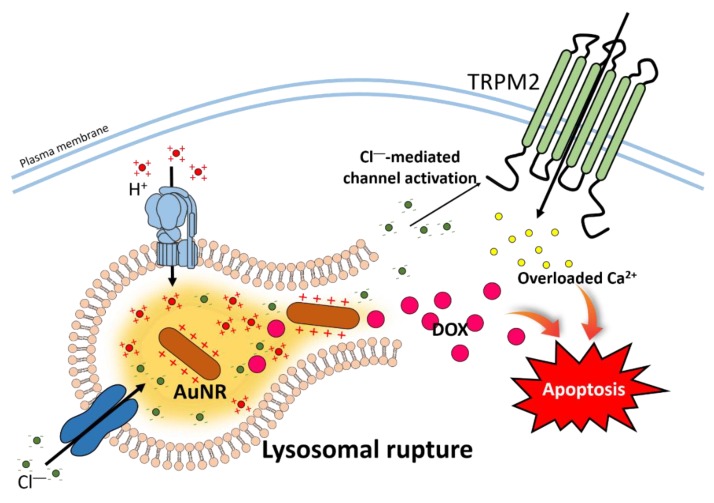
Schematic cartoon illustrating mechanism of AuNR-DOX-induced apoptosis. The hydrolysis of AuNR-DOX induces AuNR to reflect positive charge and triggers chloride influx into lysosomes. Continued chloride influx leads excessive activation of V-ATPase, and lysosomal swelling and rupture to release DOX and chloride to the cytoplasm. The DOX and Ca^2+^ through the chloride-activated TRPM2 increase cellular apoptosis. Abbreviation: V-ATPase: Vacuolar type of H^+^-ATPase.

Swelling of lysosomes has the potential to increase cellular toxicity by releasing lysosomal compartments and nanoparticles [187,188]. The lysosomal ‘proton sponge effect’ is triggered by the influx of cationic nanoparticles with hydrogen and chloride ions to lysosomes [188]. Accumulated ions in the lysosome may trigger water intake to equilibrate the physiological osmolarity and, subsequently, induce lysosomal rupture [188]. It has been addressed that conceptual use of the lysosomal pH-dependent system and lysosomal rupture develops the self-assembled luminescent AuNPs by the swelling property [189]. In a previous study, we reported that the cationic nanorod conjugated with doxorubicin (DOX) (AuNR-DOX) induced lysosomal swelling and rupture with increased apoptosis (Figure 3) [190]. Lee et al. reported that encapsulated AuNR-DOX in lysosomes is dissociated with DOX by lysosomal hydrolases. A charged linker of AuNR is opened and then recruited negative charged ions such as chloride into the lysosome. The ionic accumulation is developed, and lysosomal rupture occurred. Released chloride from the lysosome through lysosomal rupture activates Ca^2+^ influx channel TRPM2 in the plasma membrane and, lastly, overload of Ca^2+^ triggers the enhanced apoptotic effect including the effect of DOX in cancer cells [190]. The intracellular mechanism of nanomaterials and its related channels is now started. However, the effect of nanoparticles on lysosomal ion channels and transporters has still been poorly studied. To use nanomaterials for medicines, understanding the relationship between nanoparticles and lysosomal ion channels has to be expanded.

## 5. Clinical Application and Limitation of Nanomaterials

As mentioned earlier, NPs have a bio-toxic effect on lysosomes by triggering pH alteration, malfunctions of protein activity, accumulation in lysosomes, and subsequent cell death. We summarized the effect of NPs on cellular functions in Table 2. Accordingly, application of NPs has limitations for nanodrugs and nano-therapies. Thus, recent efforts have challenged to overcome these limitations by maximizing transport ability or reducing cytotoxicity.

Nanomaterials can act as the carrier for conventional drugs by transporting drugs or proteins through lysosomes such as AuNRs conjugated with Naja kaouthia protein toxin 1 (NKCT1) (one of the snake toxin protein) [191], silk NPs conjugated with doxorubicin (anti-cancer drugs) [192], and AgNPs conjugated with salinomycin (killing agent for cancer stem cells) [193]. These nanomaterials can maximize drug delivery to reach the lysosome easily, and, subsequently, kill the cancer cells from leukemia [191], breast cancer [192], and ovarian cancer [193]. The “small size” of NPs, which is one of the typical characteristics, can be used to penetrate obstacles that conventional drugs cannot cross, especially the blood brain barrier (BBB) [194]. One of the LSD, Gaucher’s type 3 disease, which occurs by accumulation of glucocerebroside in the brain can be cured by transporting enzymes into the brain [194]. A recent study demonstrated the potential for transporting enzymes across the BBB by using a recombinant arylsulfatase enzyme with polysorbate 80 coated poly-butyl cyanoacrylates NPs [195].

Although biocompatible nanodrugs have been developed, which are made of albumin-based [196,197,198] and lipid-based [199,200] nanoparticles, various studies have attempted to eliminate the toxicity of NPs via conjugation with other materials. For example, iron oxide NPs that induce autophagosome accumulation and impair lysosomes can be rendered bio-safe by coating with poly(lactic-*co*-glycolic acid) (PLGA) [193]. ZnO NPs and Quantum Dots that induce lysosomal damage with the generation of ROS can be stabilized by coating with α-linolenic acid [201] and 3-mercaptopropionic acid [202]. There are non-toxic nanomaterials that can be degraded into lysosomes, such as nano-diamonds, which are delivered to lysosomes by coating with ubiquitin, to associate with autophagy receptors: sequestosome 1 [203], Ca^2+^ binding and coiled-coil domain 2 [204,205], and optineurin [206]. Additionally, PLGA NPs are degraded easily in the autophagy pathway [207]. Adjustment of the NPs size can avoid lysosomal accumulation: 60 nm-sized TiO_2_ NPs are more aggregated and more destabilized in the lysosomal membrane than 180 nm-sized TiO_2_ in the lysosomes and endosomes [208].

## 6. Future Perspectives

**Figure 4:**
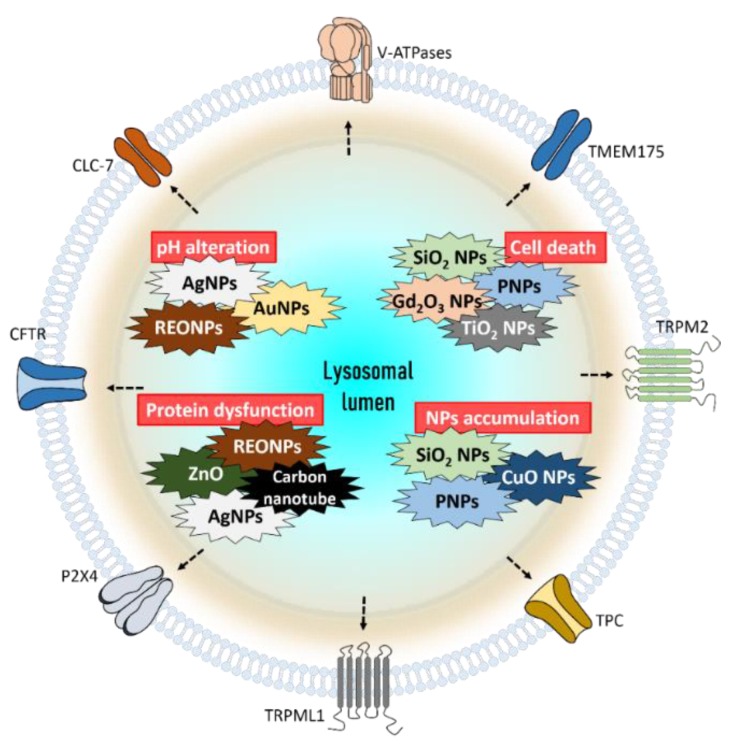
The summarized role of nanoparticles which effect lysosomes and cellular functions. Exposure to AgNPs, AuNPs, and REONPs induces alkalization of lysosomal lumen. SiO_2_ NPs, Gd_2_O_3_ NPs, PNPs, and TiO_2_ NPs damage lysosomes, which finally trigger cell death. The lysosomal protein can be damaged by ZnO, REONPs, AgNPs, and carbon nanotubes. Accumulation of SiO2 NPs, PNPs, and CuO NPs can induce lysosomal dysfunction. Abbreviations: REONP: rare earth oxide nanoparticle; PNP: polystyrene nanoparticle

The primary lysosomal function is to maintain cellular homeostasis. Various attempts of drug delivery systems including nanomaterials and other new paradigms against diseases were engaged (Figure 4). However, a plethora of questions should be answered about nano therapy against lysosomal targets or lysosomal pathways. Although our limited knowledge about the effect of nanomaterials on lysosomal function has been posted, its therapeutic potential cannot be neglected. Nanomaterials are attractive machinery, as carriers for conventional drugs for therapeutic purposes. In addition to the role of the attractive carrier, other unfavorable characteristics of nanomaterials including toxicity should be considered while developing the therapeutic strategies. Understanding the functional support of ion channels or transporters on the lysosome will be expanded further in the coming years and, subsequently, favorable potential of nanomaterial-based therapy will also improve.

## Figures and Tables

**Table 1:** The relationship between lysosomal ion channels and cellular functions.

Channels	Mechanisms and Related Diseases	Ref.
CLC-3	Promotion of lysosomal acidification	[72,73]
CLC-6	LSD in CLC-6 mutated neuronal cells	[74]
CLC-7	Maintenance of acidic pH of lysosomes	[75,76]
	Decrease of dentinogenesis and dental bone formation in CLC-7 deficient mice	[77,78]
	Degradation of fAβ which drives AD	[79,80]
	Osteopetrosis in CLC-7 mutation	[81,82,83,84]
	LSD and neurodegeneration in CLC-7-deficient mice	[64,82]
CFTR	Support lysosomal acidification	[85]
	Decrease of bacteria killing function and phago-lysosomal fusion in macrophage	[86]
TRPM2	Induce DC maturation and migration	[87]
	Increase of actin remodeling	[88]
	Increase of pancreatic β cell apoptosis	[89]
	Increase LMP, NLRP3 inflammasome, and mitochondrial fission on the plasma membrane	[90,91]
TRPML1	Maintenance of acidic pH of lysosomes	[92]
	Increase of large particle phagocytosis, bone remodeling, gastric acid secretion, and myocytes apoptosis	[93,94,95,96]
	Stomach hypertrophy, hypergastrinemia, LSD, mucolipidosis, NPC, and AD in TRPML1 deficiency	[93,97,98,99,100,101,102,103]
TMEM175	Support lysosomal Ca2+ signaling and pH regulation	[104]
	Related in LSD	[105]
TPC	Related in autophagy, cancer cell migration, and cellular pigmentation	[106,107,108]
	Related in Parkinson’s disease	[109,110]
P2X4	Promotion of endo-lysosomal fusion	[111,112]
	Related in liver fibrogenesis	[113]

Abbreviations: CLC: Chloride channel; CFTR: Cystic fibrosis transmembrane conductance regulator; TRPM2: Transient receptor potential melastatin 2; TRPML1: Transient receptor potential mucolipin 1; TMEM175: Transmembrane protein 175; TPC: Two pore channel; AD: Alzheimer’s disease; DC: dendritic cell; LMP: Lysosomal membrane permeabilization; NLRP3: NACHT, LRR and PYD domains-containing protein 3; NPC: Niemann-Pick disease type C.

**Table 2:** The effect of nanoparticles (NPs) on cellular functions.

Related Cellular Function	NPs	Details	Reference
**pH alteration** **(alkalization of lysosome)**	AuNPs	Increase of oxidative stress, mitochondrial damage, and decrease cell migration/invasion	[11]
		Accumulation of LC3 and block p62 degradation	[12]
	AgNPs	Decrease of TFEB protein expression	[13]
	REONPs	Activation of IL-1β inflammasome	[14]
**Cell viability** **(cell death)**	PNPs	Decrease of autophagic flux	[32]
		Decrease of cathepsin release	[34]
	SiO_2_ NPs	Increase of membrane damage and NLRP inflammasome	[35,44]
	TiO_2_ NPs	Increase of membrane damage	[36]
	Gd_2_O_3_ NPs	Increase of membrane damage and necrosis	[37]
**Protein activity and expression**	AgNPs	Decrease of lysosomal protease activities	[41]
	REONPs	Induce lysosomal imbalance by inhibiting mTORC1 pathway	[43]
	ZnO NPs	Increase of macrophage cell death by inhibiting mTORC1 pathway	[42]
		Deglycosylation of LAMP-2	[44]
	Carbon nanotube	Decrease of SNAP	[50]
**Accumulation of NPs**	CuO NPs	Subsequent cellular damage leading to cell death by agglomeration of lysosomes	[55,56]
	SiO_2_ NPs, PNPs	Induce lysosomal swelling leading to apoptosis	[57,58]

Abbreviations: AuNP: Gold nanoparticle; AgNP: Silver nanoparticle; REONP: rare earth oxide nanoparticle; PNP: polystyrene nanoparticle; ZnO: Zinc oxide; CuO: Copper oxide; TFEB: Transcription factor EB; IL-1β: interleukin-1β; NLRP: NACHT, LRR and PYD domains-containing protein; mTORC1: rapamycin complex 1; SNAP: synaptosomal-associated protein.

## References

[1] De Duve C., Wattiaux R. (1966). Functions of lysosomes. Annu. Rev. Physiol..

[2] de Duve C. (2005). The lysosome turns fifty. Nat. Cell Biol..

[3] Perera R.M., Zoncu R. (2016). The Lysosome as a Regulatory Hub. Annu. Rev. Cell Dev. Biol..

[4] Pu J., Guardia C.M., Keren-Kaplan T., Bonifacino J.S. (2016). Mechanisms and functions of lysosome positioning. J. Cell Sci..

[5] Mindell J.A. (2012). Lysosomal acidification mechanisms. Annu. Rev. Physiol..

[6] Walkley S.U. (2007). Pathogenic mechanisms in lysosomal disease: A reappraisal of the role of the lysosome. Acta Paediatr..

[7] Hipolito V.E.B., Ospina-Escobar E., Botelho R.J. (2018). Lysosome remodelling and adaptation during phagocyte activation. Cell Microbiol..

[8] Herb M., Gluschko A., Schramm M. (2019). LC3-associated phagocytosis—The highway to hell for phagocytosed microbes. Semin. Cell Dev. Biol..

[9] Folts C.J., Scott-Hewitt N., Proschel C., Mayer-Proschel M., Noble M. (2016). Lysosomal Re-acidification Prevents Lysosphingolipid-Induced Lysosomal Impairment and Cellular Toxicity. PLoS Biol..

[10] Stern S.T., Adiseshaiah P.P., Crist R.M. (2012). Autophagy and lysosomal dysfunction as emerging mechanisms of nanomaterial toxicity. Part. Fibre Toxicol..

[11] Woldemichael T., Rosania G.R. (2017). The physiological determinants of drug-induced lysosomal stress resistance. PLoS ONE.

[12] Ma X., Wu Y., Jin S., Tian Y., Zhang X., Zhao Y., Yu L., Liang X.J. (2011). Gold nanoparticles induce autophagosome accumulation through size-dependent nanoparticle uptake and lysosome impairment. ACS Nano.

[13] Miyayama T., Fujiki K., Matsuoka M. (2018). Silver nanoparticles induce lysosomal-autophagic defects and decreased expression of transcription factor EB in A549 human lung adenocarcinoma cells. Toxicol. Vitr..

[14] Li R., Ji Z., Qin H., Kang X., Sun B., Wang M., Chang C.H., Wang X., Zhang H., Zou H. (2014). Interference in autophagosome fusion by rare earth nanoparticles disrupts autophagic flux and regulation of an interleukin-1beta producing inflammasome. ACS Nano.

[15] Winchester B.G. (2001). Lysosomal membrane proteins. Eur. J. Paediatr. Neurol..

[16] Schwake M., Schroder B., Saftig P. (2013). Lysosomal membrane proteins and their central role in physiology. Traffic.

[17] Yu S., Melia T.J. (2017). The coordination of membrane fission and fusion at the end of autophagosome maturation. Curr. Opin. Cell Biol..

[18] Nascimbeni A.C., Codogno P., Morel E. (2017). Phosphatidylinositol-3-phosphate in the regulation of autophagy membrane dynamics. FEBS J..

[19] Wong Y.C., Kim S., Peng W., Krainc D. (2019). Regulation and Function of Mitochondria-Lysosome Membrane Contact Sites in Cellular Homeostasis. Trends Cell Biol..

[20] Helle S.C., Kanfer G., Kolar K., Lang A., Michel A.H., Kornmann B. (2013). Organization and function of membrane contact sites. Biochim. Biophys. Acta.

[21] Zoncu R., Bar-Peled L., Efeyan A., Wang S., Sancak Y., Sabatini D.M. (2011). mTORC1 senses lysosomal amino acids through an inside-out mechanism that requires the vacuolar H(+)-ATPase. Science.

[22] Johnson D.E., Ostrowski P., Jaumouille V., Grinstein S. (2016). The position of lysosomes within the cell determines their luminal pH. J. Cell Biol..

[23] Li P., Gu M., Xu H. (2019). Lysosomal Ion Channels as Decoders of Cellular Signals. Trends Biochem. Sci..

[24] Nishino I., Fu J., Tanji K., Yamada T., Shimojo S., Koori T., Mora M., Riggs J.E., Oh S.J., Koga Y. (2000). Primary LAMP-2 deficiency causes X-linked vacuolar cardiomyopathy and myopathy (Danon disease). Nature.

[25] Kornak U., Kasper D., Bosl M.R., Kaiser E., Schweizer M., Schulz A., Friedrich W., Delling G., Jentsch T.J. (2001). Loss of the ClC-7 chloride channel leads to osteopetrosis in mice and man. Cell.

[26] Berkovic S.F., Dibbens L.M., Oshlack A., Silver J.D., Katerelos M., Vears D.F., Lullmann-Rauch R., Blanz J., Zhang K.W., Stankovich J. (2008). Array-based gene discovery with three unrelated subjects shows SCARB2/LIMP-2 deficiency causes myoclonus epilepsy and glomerulosclerosis. Am. J. Hum. Genet..

[27] Wang F., Gomez-Sintes R., Boya P. (2018). Lysosomal membrane permeabilization and cell death. Traffic.

[28] Mrschtik M., Ryan K.M. (2015). Lysosomal proteins in cell death and autophagy. FEBS J..

[29] Serrano-Puebla A., Boya P. (2016). Lysosomal membrane permeabilization in cell death: New evidence and implications for health and disease. Ann. N. Y. Acad. Sci..

[30] Micsenyi M.C., Sikora J., Stephney G., Dobrenis K., Walkley S.U. (2013). Lysosomal membrane permeability stimulates protein aggregate formation in neurons of a lysosomal disease. J. Neurosci..

[31] Venkatesan R., Park Y.U., Ji E., Yeo E.J., Kim S.Y. (2017). Malathion increases apoptotic cell death by inducing lysosomal membrane permeabilization in N2a neuroblastoma cells: A model for neurodegeneration in Alzheimer’s disease. Cell Death Discov..

[32] Song W., Popp L., Yang J., Kumar A., Gangoli V.S., Segatori L. (2015). The autophagic response to polystyrene nanoparticles is mediated by transcription factor EB and depends on surface charge. J. Nanobiotechnol..

[33] Lunova M., Prokhorov A., Jirsa M., Hof M., Olzynska A., Jurkiewicz P., Kubinova S., Lunov O., Dejneka A. (2017). Nanoparticle core stability and surface functionalization drive the mTOR signaling pathway in hepatocellular cell lines. Sci. Rep..

[34] Wang F., Salvati A., Boya P. (2018). Lysosome-dependent cell death and deregulated autophagy induced by amine-modified polystyrene nanoparticles. Open Biol..

[35] Jessop F., Hamilton R.F., Rhoderick J.F., Fletcher P., Holian A. (2017). Phagolysosome acidification is required for silica and engineered nanoparticle-induced lysosome membrane permeabilization and resultant NLRP3 inflammasome activity. Toxicol. Appl. Pharm..

[36] Popp L., Tran V., Patel R., Segatori L. (2018). Autophagic response to cellular exposure to titanium dioxide nanoparticles. Acta Biomater..

[37] Jin Y., Chen S., Duan J., Jia G., Zhang J. (2015). Europium-doped Gd2O3 nanotubes cause the necrosis of primary mouse bone marrow stromal cells through lysosome and mitochondrion damage. J. Inorg. Biochem..

[38] Korolchuk V.I., Saiki S., Lichtenberg M., Siddiqi F.H., Roberts E.A., Imarisio S., Jahreiss L., Sarkar S., Futter M., Menzies F.M. (2011). Lysosomal positioning coordinates cellular nutrient responses. Nat. Cell Biol..

[39] Noda T., Ohsumi Y. (1998). Tor, a phosphatidylinositol kinase homologue, controls autophagy in yeast. J. Biol. Chem..

[40] Eskelinen E.L. (2006). Roles of LAMP-1 and LAMP-2 in lysosome biogenesis and autophagy. Mol. Asp. Med..

[41] Chen Y., Wang M., Zhang T., Du E., Liu Y., Qi S., Xu Y., Zhang Z. (2018). Autophagic effects and mechanisms of silver nanoparticles in renal cells under low dose exposure. Ecotoxicol. Environ. Saf..

[42] Roy R., Singh S.K., Chauhan L.K., Das M., Tripathi A., Dwivedi P.D. (2014). Zinc oxide nanoparticles induce apoptosis by enhancement of autophagy via PI3K/Akt/mTOR inhibition. Toxicol. Lett..

[43] Lin J., Shi S.S., Zhang J.Q., Zhang Y.J., Zhang L., Liu Y., Jin P.P., Wei P.F., Shi R.H., Zhou W. (2016). Giant Cellular Vacuoles Induced by Rare Earth Oxide Nanoparticles are Abnormally Enlarged Endo/Lysosomes and Promote mTOR-Dependent TFEB Nucleus Translocation. Small.

[44] Qin X., Zhang J., Wang B., Xu G., Zou Z. (2017). LAMP-2 mediates oxidative stress-dependent cell death in Zn(2+)-treated lung epithelium cells. Biochem. Biophys. Res. Commun..

[45] Cohignac V., Landry M.J., Ridoux A., Pinault M., Annangi B., Gerdil A., Herlin-Boime N., Mayne M., Haruta M., Codogno P. (2018). Carbon nanotubes, but not spherical nanoparticles, block autophagy by a shape-related targeting of lysosomes in murine macrophages. Autophagy.

[46] Hirokawa N., Noda Y. (2008). Intracellular transport and kinesin superfamily proteins, KIFs: Structure, function, and dynamics. Physiol. Rev..

[47] Hollenbeck P.J., Swanson J.A. (1990). Radial extension of macrophage tubular lysosomes supported by kinesin. Nature.

[48] Paschal B.M., Vallee R.B. (1987). Retrograde transport by the microtubule-associated protein MAP 1C. Nature.

[49] Harada A., Takei Y., Kanai Y., Tanaka Y., Nonaka S., Hirokawa N. (1998). Golgi vesiculation and lysosome dispersion in cells lacking cytoplasmic dynein. J. Cell Biol..

[50] Yuzaki M. (2010). Snapin snaps into the dynein complex for late endosome-lysosome trafficking and autophagy. Neuron.

[51] Chun Y., Kim J. (2018). Autophagy: An Essential Degradation Program for Cellular Homeostasis and Life. Cells.

[52] Settembre C., Ballabio A. (2014). Lysosome: Regulator of lipid degradation pathways. Trends Cell Biol..

[53] Lawrence R.E., Zoncu R. (2019). The lysosome as a cellular centre for signalling, metabolism and quality control. Nat. Cell Biol..

[54] Ferguson S.M. (2019). Neuronal lysosomes. Neurosci. Lett..

[55] Miyayama T., Matsuoka M. (2016). Involvement of lysosomal dysfunction in silver nanoparticle-induced cellular damage in A549 human lung alveolar epithelial cells. J. Occup. Med. Toxicol..

[56] Zhang J., Zou Z., Wang B., Xu G., Wu Q., Zhang Y., Yuan Z., Yang X., Yu C. (2018). Lysosomal deposition of copper oxide nanoparticles triggers HUVEC cells death. Biomaterials.

[57] Schutz I., Lopez-Hernandez T., Gao Q., Puchkov D., Jabs S., Nordmeyer D., Schmudde M., Ruhl E., Graf C.M., Haucke V. (2016). Lysosomal Dysfunction Caused by Cellular Accumulation of Silica Nanoparticles. J. Biol. Chem..

[58] Wang F., Bexiga M.G., Anguissola S., Boya P., Simpson J.C., Salvati A., Dawson K.A. (2013). Time resolved study of cell death mechanisms induced by amine-modified polystyrene nanoparticles. Nanoscale.

[59] Ohkuma S., Moriyama Y., Takano T. (1982). Identification and characterization of a proton pump on lysosomes by fluorescein-isothiocyanate-dextran fluorescence. Proc. Natl. Acad. Sci. USA.

[60] Cipriano D.J., Wang Y., Bond S., Hinton A., Jefferies K.C., Qi J., Forgac M. (2008). Structure and regulation of the vacuolar ATPases. Biochim. Biophys. Acta.

[61] Hirata T., Iwamoto-Kihara A., Sun-Wada G.H., Okajima T., Wada Y., Futai M. (2003). Subunit rotation of vacuolar-type proton pumping ATPase: Relative rotation of the G and C subunits. J. Biol. Chem..

[62] Yokoyama K., Nakano M., Imamura H., Yoshida M., Tamakoshi M. (2003). Rotation of the proteolipid ring in the V-ATPase. J. Biol. Chem..

[63] Ishida Y., Nayak S., Mindell J.A., Grabe M. (2013). A model of lysosomal pH regulation. J. Gen. Physiol..

[64] Kasper D., Planells-Cases R., Fuhrmann J.C., Scheel O., Zeitz O., Ruether K., Schmitt A., Poet M., Steinfeld R., Schweizer M. (2005). Loss of the chloride channel ClC-7 leads to lysosomal storage disease and neurodegeneration. EMBO J..

[65] Checchetto V., Teardo E., Carraretto L., Leanza L., Szabo I. (2016). Physiology of intracellular potassium channels: A unifying role as mediators of counterion fluxes?. Biochim. Biophys. Acta.

[66] Betz C., Hall M.N. (2013). Where is mTOR and what is it doing there?. J. Cell Biol..

[67] Sundler R. (1997). Lysosomal and cytosolic pH as regulators of exocytosis in mouse macrophages. Acta Physiol. Scand..

[68] Tapper H., Sundler R. (1990). Role of lysosomal and cytosolic pH in the regulation of macrophage lysosomal enzyme secretion. Biochem. J..

[69] Camargo M.J., Sumpio B.E., Maack T. (1984). Renal hydrolysis of absorbed protein: Influence of load and lysosomal pH. Am. J. Physiol..

[70] Smith M.L., Greene A.A., Potashnik R., Mendoza S.A., Schneider J.A. (1987). Lysosomal cystine transport. Effect of intralysosomal pH and membrane potential. J. Biol. Chem..

[71] Lee D., Hong J.H. (2019). Physiological application of nanoparticles in calcium-related proteins and channels. Nanomed..

[72] Li X., Wang T., Zhao Z., Weinman S.A. (2002). The ClC-3 chloride channel promotes acidification of lysosomes in CHO-K1 and Huh-7 cells. Am. J. Physiol. Cell Physiol..

[73] Okamoto F., Kajiya H., Toh K., Uchida S., Yoshikawa M., Sasaki S., Kido M.A., Tanaka T., Okabe K. (2008). Intracellular ClC-3 chloride channels promote bone resorption in vitro through organelle acidification in mouse osteoclasts. Am. J. Physiol. Cell Physiol..

[74] Poet M., Kornak U., Schweizer M., Zdebik A.A., Scheel O., Hoelter S., Wurst W., Schmitt A., Fuhrmann J.C., Planells-Cases R. (2006). Lysosomal storage disease upon disruption of the neuronal chloride transport protein ClC-6. Proc. Natl. Acad. Sci. USA.

[75] Henriksen K., Gram J., Neutzsky-Wulff A.V., Jensen V.K., Dziegiel M.H., Bollerslev J., Karsdal M.A. (2009). Characterization of acid flux in osteoclasts from patients harboring a G215R mutation in ClC-7. Biochem. Biophys. Res. Commun..

[76] Henriksen K., Sorensen M.G., Jensen V.K., Dziegiel M.H., Nosjean O., Karsdal M.A. (2008). Ion transporters involved in acidification of the resorption lacuna in osteoclasts. Calcif. Tissue Int..

[77] Wen X., Lacruz R.S., Paine M.L. (2015). Dental and Cranial Pathologies in Mice Lacking the Cl(-) /H(+) -Exchanger ClC-7. Anat. Rec..

[78] Guo J., Bervoets T.J., Henriksen K., Everts V., Bronckers A.L. (2016). Null mutation of chloride channel 7 (Clcn7) impairs dental root formation but does not affect enamel mineralization. Cell Tissue Res..

[79] Murphy M.P., LeVine H. (2010). Alzheimer’s disease and the amyloid-beta peptide. J. Alzheimers Dis..

[80] Majumdar A., Capetillo-Zarate E., Cruz D., Gouras G.K., Maxfield F.R. (2011). Degradation of Alzheimer’s amyloid fibrils by microglia requires delivery of ClC-7 to lysosomes. Mol. Biol. Cell.

[81] Jentsch T.J. (2007). Chloride and the endosomal-lysosomal pathway: Emerging roles of CLC chloride transporters. J. Physiol..

[82] Zhao Q., Wei Q., He A., Jia R., Xiao Y. (2009). CLC-7: A potential therapeutic target for the treatment of osteoporosis and neurodegeneration. Biochem. Biophys. Res. Commun..

[83] Weinert S., Jabs S., Supanchart C., Schweizer M., Gimber N., Richter M., Rademann J., Stauber T., Kornak U., Jentsch T.J. (2010). Lysosomal pathology and osteopetrosis upon loss of H+-driven lysosomal Cl- accumulation. Science.

[84] Sartelet A., Stauber T., Coppieters W., Ludwig C.F., Fasquelle C., Druet T., Zhang Z., Ahariz N., Cambisano N., Jentsch T.J. (2014). A missense mutation accelerating the gating of the lysosomal Cl-/H+-exchanger ClC-7/Ostm1 causes osteopetrosis with gingival hamartomas in cattle. Dis. Model. Mech..

[85] Haggie P.M., Verkman A.S. (2009). Unimpaired lysosomal acidification in respiratory epithelial cells in cystic fibrosis. J. Biol. Chem..

[86] Di A., Brown M.E., Deriy L.V., Li C., Szeto F.L., Chen Y., Huang P., Tong J., Naren A.P., Bindokas V. (2006). CFTR regulates phagosome acidification in macrophages and alters bactericidal activity. Nat. Cell Biol..

[87] Sumoza-Toledo A., Lange I., Cortado H., Bhagat H., Mori Y., Fleig A., Penner R., Partida-Sanchez S. (2011). Dendritic cell maturation and chemotaxis is regulated by TRPM2-mediated lysosomal Ca2+ release. FASEB J..

[88] Li F., Abuarab N., Sivaprasadarao A. (2016). Reciprocal regulation of actin cytoskeleton remodelling and cell migration by Ca2+ and Zn2+: Role of TRPM2 channels. J. Cell Sci..

[89] Lange I., Yamamoto S., Partida-Sanchez S., Mori Y., Fleig A., Penner R. (2009). TRPM2 functions as a lysosomal Ca2+-release channel in beta cells. Sci. Signal..

[90] Abuarab N., Munsey T.S., Jiang L.H., Li J., Sivaprasadarao A. (2017). High glucose-induced ROS activates TRPM2 to trigger lysosomal membrane permeabilization and Zn(2+)-mediated mitochondrial fission. Sci. Signal..

[91] Katsnelson M.A., Lozada-Soto K.M., Russo H.M., Miller B.A., Dubyak G.R. (2016). NLRP3 inflammasome signaling is activated by low-level lysosome disruption but inhibited by extensive lysosome disruption: Roles for K+ efflux and Ca2+ influx. Am. J. Physiol. Cell Physiol..

[92] Soyombo A.A., Tjon-Kon-Sang S., Rbaibi Y., Bashllari E., Bisceglia J., Muallem S., Kiselyov K. (2006). TRP-ML1 regulates lysosomal pH and acidic lysosomal lipid hydrolytic activity. J. Biol. Chem..

[93] Chandra M., Zhou H., Li Q., Muallem S., Hofmann S.L., Soyombo A.A. (2011). A role for the Ca2+ channel TRPML1 in gastric acid secretion, based on analysis of knockout mice. Gastroenterology.

[94] Erkhembaatar M., Gu D.R., Lee S.H., Yang Y.M., Park S., Muallem S., Shin D.M., Kim M.S. (2017). Lysosomal Ca2+ Signaling is Essential for Osteoclastogenesis and Bone Remodeling. J. Bone Min. Res..

[95] Xu M., Li X., Walsh S.W., Zhang Y., Abais J.M., Boini K.M., Li P.L. (2013). Intracellular two-phase Ca2+ release and apoptosis controlled by TRP-ML1 channel activity in coronary arterial myocytes. Am. J. Physiol. Cell Physiol..

[96] Samie M., Wang X., Zhang X., Goschka A., Li X., Cheng X., Gregg E., Azar M., Zhuo Y., Garrity A.G. (2013). A TRP channel in the lysosome regulates large particle phagocytosis via focal exocytosis. Dev. Cell.

[97] Shen D., Wang X., Li X., Zhang X., Yao Z., Dibble S., Dong X.P., Yu T., Lieberman A.P., Showalter H.D. (2012). Lipid storage disorders block lysosomal trafficking by inhibiting a TRP channel and lysosomal calcium release. Nat. Commun..

[98] Weiss N. (2012). Cross-talk between TRPML1 channel, lipids and lysosomal storage diseases. Commun. Integr. Biol..

[99] Zeevi D.A., Frumkin A., Bach G. (2007). TRPML and lysosomal function. Biochim. Biophys. Acta.

[100] Dong X.P., Cheng X., Mills E., Delling M., Wang F., Kurz T., Xu H. (2008). The type IV mucolipidosis-associated protein TRPML1 is an endolysosomal iron release channel. Nature.

[101] Miedel M.T., Rbaibi Y., Guerriero C.J., Colletti G., Weixel K.M., Weisz O.A., Kiselyov K. (2008). Membrane traffic and turnover in TRP-ML1-deficient cells: A revised model for mucolipidosis type IV pathogenesis. J. Exp. Med..

[102] Zhang F., Jin S., Yi F., Li P.L. (2009). TRP-ML1 functions as a lysosomal NAADP-sensitive Ca2+ release channel in coronary arterial myocytes. J. Cell Mol. Med..

[103] Lee J.H., McBrayer M.K., Wolfe D.M., Haslett L.J., Kumar A., Sato Y., Lie P.P., Mohan P., Coffey E.E., Kompella U. (2015). Presenilin 1 Maintains Lysosomal Ca(2+) Homeostasis via TRPML1 by Regulating vATPase-Mediated Lysosome Acidification. Cell Rep..

[104] Sterea A.M., Almasi S., El Hiani Y. (2018). The hidden potential of lysosomal ion channels: A new era of oncogenes. Cell Calcium..

[105] Feng X., Zhao Z., Li Q., Tan Z. (2018). Lysosomal Potassium Channels: Potential Roles in Lysosomal Function and Neurodegenerative Diseases. CNS Neurol. Disord. Drug Targets.

[106] Sun W., Yue J. (2018). TPC2 mediates autophagy progression and extracellular vesicle secretion in cancer cells. Exp. Cell Res..

[107] Grimm C., Bartel K., Vollmar A.M., Biel M. (2018). Endolysosomal Cation Channels and Cancer-A Link with Great Potential. Pharmaceuticals.

[108] Lin-Moshier Y., Keebler M.V., Hooper R., Boulware M.J., Liu X., Churamani D., Abood M.E., Walseth T.F., Brailoiu E., Patel S. (2014). The Two-pore channel (TPC) interactome unmasks isoform-specific roles for TPCs in endolysosomal morphology and cell pigmentation. Proc. Natl. Acad. Sci. USA.

[109] Hockey L.N., Kilpatrick B.S., Eden E.R., Lin-Moshier Y., Brailoiu G.C., Brailoiu E., Futter C.E., Schapira A.H., Marchant J.S., Patel S. (2015). Dysregulation of lysosomal morphology by pathogenic LRRK2 is corrected by TPC2 inhibition. J. Cell Sci..

[110] Rivero-Rios P., Gomez-Suaga P., Fernandez B., Madero-Perez J., Schwab A.J., Ebert A.D., Hilfiker S. (2015). Alterations in late endocytic trafficking related to the pathobiology of LRRK2-linked Parkinson’s disease. Biochem Soc. Trans..

[111] Cao Q., Zhong X.Z., Zou Y., Murrell-Lagnado R., Zhu M.X., Dong X.P. (2015). Calcium release through P2X4 activates calmodulin to promote endolysosomal membrane fusion. J. Cell Biol..

[112] Fois G., Winkelmann V.E., Bareis L., Staudenmaier L., Hecht E., Ziller C., Ehinger K., Schymeinsky J., Kranz C., Frick M. (2018). ATP is stored in lamellar bodies to activate vesicular P2X4 in an autocrine fashion upon exocytosis. J. Gen. Physiol..

[113] Le Guilcher C., Garcin I., Dellis O., Cauchois F., Tebbi A., Doignon I., Guettier C., Julien B., Tordjmann T. (2018). The P2X4 purinergic receptor regulates hepatic myofibroblast activation during liver fibrogenesis. J. Hepatol..

[114] Poroca D.R., Pelis R.M., Chappe V.M. (2017). ClC Channels and Transporters: Structure, Physiological Functions, and Implications in Human Chloride Channelopathies. Front. Pharm..

[115] Stauber T., Jentsch T.J. (2010). Sorting motifs of the endosomal/lysosomal CLC chloride transporters. J. Biol. Chem..

[116] Zifarelli G. (2015). A tale of two CLCs: Biophysical insights toward understanding ClC-5 and ClC-7 function in endosomes and lysosomes. J. Physiol..

[117] Graves A.R., Curran P.K., Smith C.L., Mindell J.A. (2008). The Cl-/H+ antiporter ClC-7 is the primary chloride permeation pathway in lysosomes. Nature.

[118] Braun A.P. (2008). Identification of ClC-7 as a major pathway for Cl- movement in lysosomes. Channels.

[119] Lange P.F., Wartosch L., Jentsch T.J., Fuhrmann J.C. (2006). ClC-7 requires Ostm1 as a beta-subunit to support bone resorption and lysosomal function. Nature.

[120] Fuller C.M., Benos D.J. (1992). Cftr!. Am. J. Physiol..

[121] Meng X., Clews J., Kargas V., Wang X., Ford R.C. (2017). The cystic fibrosis transmembrane conductance regulator (CFTR) and its stability. Cell Mol. Life Sci..

[122] Moran O. (2017). The gating of the CFTR channel. Cell Mol. Life Sci..

[123] Borowitz D. (2015). CFTR, bicarbonate, and the pathophysiology of cystic fibrosis. Pediatr. Pulmonol..

[124] Csanady L., Vergani P., Gadsby D.C. (2019). Structure, Gating, and Regulation of the Cftr Anion Channel. Physiol. Rev..

[125] Riordan J.R. (2008). CFTR function and prospects for therapy. Annu. Rev. Biochem..

[126] Bradbury N.A. (1999). Intracellular CFTR: Localization and function. Physiol. Rev..

[127] Swanson J. (2006). CFTR: Helping to acidify macrophage lysosomes. Nat. Cell Biol..

[128] Liu J., Lu W., Guha S., Baltazar G.C., Coffey E.E., Laties A.M., Rubenstein R.C., Reenstra W.W., Mitchell C.H. (2012). Cystic fibrosis transmembrane conductance regulator contributes to reacidification of alkalinized lysosomes in RPE cells. Am. J. Physiol. Cell Physiol..

[129] Clapham D.E., Runnels L.W., Strubing C. (2001). The TRP ion channel family. Nat. Rev. Neurosci..

[130] Ramsey I.S., Delling M., Clapham D.E. (2006). An introduction to TRP channels. Annu. Rev. Physiol..

[131] Bootman M.D., Chehab T., Bultynck G., Parys J.B., Rietdorf K. (2018). The regulation of autophagy by calcium signals: Do we have a consensus?. Cell Calcium..

[132] Sano Y., Inamura K., Miyake A., Mochizuki S., Yokoi H., Matsushime H., Furuichi K. (2001). Immunocyte Ca2+ influx system mediated by LTRPC2. Science.

[133] Perraud A.L., Fleig A., Dunn C.A., Bagley L.A., Launay P., Schmitz C., Stokes A.J., Zhu Q., Bessman M.J., Penner R. (2001). ADP-ribose gating of the calcium-permeable LTRPC2 channel revealed by Nudix motif homology. Nature.

[134] Fleig A., Penner R. (2004). Emerging roles of TRPM channels. Novartis. Found. Symp..

[135] Wehage E., Eisfeld J., Heiner I., Jungling E., Zitt C., Luckhoff A. (2002). Activation of the cation channel long transient receptor potential channel 2 (LTRPC2) by hydrogen peroxide. A splice variant reveals a mode of activation independent of ADP-ribose. J. Biol. Chem..

[136] Hara Y., Wakamori M., Ishii M., Maeno E., Nishida M., Yoshida T., Yamada H., Shimizu S., Mori E., Kudoh J. (2002). LTRPC2 Ca2+-permeable channel activated by changes in redox status confers susceptibility to cell death. Mol. Cell.

[137] Kolisek M., Beck A., Fleig A., Penner R. (2005). Cyclic ADP-ribose and hydrogen peroxide synergize with ADP-ribose in the activation of TRPM2 channels. Mol. Cell.

[138] McHugh D., Flemming R., Xu S.Z., Perraud A.L., Beech D.J. (2003). Critical intracellular Ca2+ dependence of transient receptor potential melastatin 2 (TRPM2) cation channel activation. J. Biol. Chem..

[139] Starkus J., Beck A., Fleig A., Penner R. (2007). Regulation of TRPM2 by extra- and intracellular calcium. J. Gen. Physiol..

[140] Csanady L., Torocsik B. (2009). Four Ca2+ ions activate TRPM2 channels by binding in deep crevices near the pore but intracellularly of the gate. J. Gen. Physiol..

[141] Perraud A.L., Schmitz C., Scharenberg A.M. (2003). TRPM2 Ca2+ permeable cation channels: From gene to biological function. Cell Calcium..

[142] Sumoza-Toledo A., Penner R. (2011). TRPM2: A multifunctional ion channel for calcium signalling. J. Physiol.

[143] Takahashi N., Kozai D., Kobayashi R., Ebert M., Mori Y. (2011). Roles of TRPM2 in oxidative stress. Cell Calcium..

[144] Puertollano R., Kiselyov K. (2009). TRPMLs: In sickness and in health. Am. J. Physiol Ren. Physiol..

[145] Qian F., Noben-Trauth K. (2005). Cellular and molecular function of mucolipins (TRPML) and polycystin 2 (TRPP2). Pflug. Arch..

[146] Dong X.P., Wang X., Xu H. (2010). TRP channels of intracellular membranes. J. Neurochem..

[147] Abe K., Puertollano R. (2011). Role of TRP channels in the regulation of the endosomal pathway. Physiology.

[148] Grimm C., Hassan S., Wahl-Schott C., Biel M. (2012). Role of TRPML and two-pore channels in endolysosomal cation homeostasis. J. Pharm. Exp..

[149] Venkatachalam K., Wong C.O., Zhu M.X. (2015). The role of TRPMLs in endolysosomal trafficking and function. Cell Calcium.

[150] Patel S., Cai X. (2015). Evolution of acidic Ca(2)(+) stores and their resident Ca(2)(+)-permeable channels. Cell Calcium.

[151] Zhang X., Hu M., Yang Y., Xu H. (2018). Organellar TRP channels. Nat. Struct. Mol. Biol..

[152] Karacsonyi C., Miguel A.S., Puertollano R. (2007). Mucolipin-2 localizes to the Arf6-associated pathway and regulates recycling of GPI-APs. Traffic.

[153] Sun L., Hua Y., Vergarajauregui S., Diab H.I., Puertollano R. (2015). Novel Role of TRPML2 in the Regulation of the Innate Immune Response. J. Immunol..

[154] Lindvall J.M., Blomberg K.E., Valiaho J., Vargas L., Heinonen J.E., Berglof A., Mohamed A.J., Nore B.F., Vihinen M., Smith C.I. (2005). Bruton’s tyrosine kinase: Cell biology, sequence conservation, mutation spectrum, siRNA modifications, and expression profiling. Immunol. Rev..

[155] Miao Y., Li G., Zhang X., Xu H., Abraham S.N. (2015). A TRP Channel Senses Lysosome Neutralization by Pathogens to Trigger Their Expulsion. Cell.

[156] Guo Z., Grimm C., Becker L., Ricci A.J., Heller S. (2013). A novel ion channel formed by interaction of TRPML3 with TRPV5. PLoS ONE.

[157] Di Palma F., Belyantseva I.A., Kim H.J., Vogt T.F., Kachar B., Noben-Trauth K. (2002). Mutations in Mcoln3 associated with deafness and pigmentation defects in varitint-waddler (Va) mice. Proc. Natl. Acad. Sci. USA.

[158] Kim H.J., Soyombo A.A., Tjon-Kon-Sang S., So I., Muallem S. (2009). The Ca(2+) channel TRPML3 regulates membrane trafficking and autophagy. Traffic.

[159] Medina D.L., Di Paola S., Peluso I., Armani A., De Stefani D., Venditti R., Montefusco S., Scotto-Rosato A., Prezioso C., Forrester A. (2015). Lysosomal calcium signalling regulates autophagy through calcineurin and TFEB. Nat. Cell Biol..

[160] Settembre C., Di Malta C., Polito V.A., Garcia Arencibia M., Vetrini F., Erdin S., Erdin S.U., Huynh T., Medina D., Colella P. (2011). TFEB links autophagy to lysosomal biogenesis. Science.

[161] Scotto Rosato A., Montefusco S., Soldati C., Di Paola S., Capuozzo A., Monfregola J., Polishchuk E., Amabile A., Grimm C., Lombardo A. (2019). TRPML1 links lysosomal calcium to autophagosome biogenesis through the activation of the CaMKKbeta/VPS34 pathway. Nat. Commun..

[162] Capurro M.I., Greenfield L.K., Prashar A., Xia S., Abdullah M., Wong H., Zhong X.Z., Bertaux-Skeirik N., Chakrabarti J., Siddiqui I. (2019). VacA generates a protective intracellular reservoir for Helicobacter pylori that is eliminated by activation of the lysosomal calcium channel TRPML1. Nat. Microbiol..

[163] Qi X., Man S.M., Malireddi R.K., Karki R., Lupfer C., Gurung P., Neale G., Guy C.S., Lamkanfi M., Kanneganti T.D. (2016). Cathepsin B modulates lysosomal biogenesis and host defense against Francisella novicida infection. J. Exp. Med..

[164] Cao Q., Yang Y., Zhong X.Z., Dong X.P. (2017). The lysosomal Ca(2+) release channel TRPML1 regulates lysosome size by activating calmodulin. J. Biol. Chem..

[165] Cang C., Aranda K., Seo Y.J., Gasnier B., Ren D. (2015). TMEM175 Is an Organelle K(+) Channel Regulating Lysosomal Function. Cell.

[166] Jinn S., Drolet R.E., Cramer P.E., Wong A.H., Toolan D.M., Gretzula C.A., Voleti B., Vassileva G., Disa J., Tadin-Strapps M. (2017). TMEM175 deficiency impairs lysosomal and mitochondrial function and increases alpha-synuclein aggregation. Proc. Natl. Acad. Sci. USA.

[167] Tugba Durlu-Kandilci N., Ruas M., Chuang K.T., Brading A., Parrington J., Galione A. (2010). TPC2 proteins mediate nicotinic acid adenine dinucleotide phosphate (NAADP)- and agonist-evoked contractions of smooth muscle. J. Biol. Chem..

[168] Zhu M.X., Evans A.M., Ma J., Parrington J., Galione A. (2010). Two-pore channels for integrative Ca signaling. Commun. Integr. Biol..

[169] Grimm C., Butz E., Chen C.C., Wahl-Schott C., Biel M. (2017). From mucolipidosis type IV to Ebola: TRPML and two-pore channels at the crossroads of endo-lysosomal trafficking and disease. Cell Calcium..

[170] Calcraft P.J., Ruas M., Pan Z., Cheng X., Arredouani A., Hao X., Tang J., Rietdorf K., Teboul L., Chuang K.T. (2009). NAADP mobilizes calcium from acidic organelles through two-pore channels. Nature.

[171] Wang X., Zhang X., Dong X.P., Samie M., Li X., Cheng X., Goschka A., Shen D., Zhou Y., Harlow J. (2012). TPC proteins are phosphoinositide- activated sodium-selective ion channels in endosomes and lysosomes. Cell.

[172] Cang C., Zhou Y., Navarro B., Seo Y.J., Aranda K., Shi L., Battaglia-Hsu S., Nissim I., Clapham D.E., Ren D. (2013). mTOR regulates lysosomal ATP-sensitive two-pore Na(+) channels to adapt to metabolic state. Cell.

[173] Ogunbayo O.A., Zhu Y., Shen B., Agbani E., Li J., Ma J., Zhu M.X., Evans A.M. (2015). Organelle-specific subunit interactions of the vertebrate two-pore channel family. J. Biol. Chem..

[174] Cang C., Aranda K., Ren D. (2014). A non-inactivating high-voltage-activated two-pore Na(+) channel that supports ultra-long action potentials and membrane bistability. Nat. Commun..

[175] Ruas M., Davis L.C., Chen C.C., Morgan A.J., Chuang K.T., Walseth T.F., Grimm C., Garnham C., Powell T., Platt N. (2015). Expression of Ca(2)(+)-permeable two-pore channels rescues NAADP signalling in TPC-deficient cells. EMBO J..

[176] Patel S. (2015). Function and dysfunction of two-pore channels. Sci. Signal..

[177] Marchant J.S., Patel S. (2015). Two-pore channels at the intersection of endolysosomal membrane traffic. Biochem Soc. Trans..

[178] Grimm C., Chen C.C., Wahl-Schott C., Biel M. (2017). Two-Pore Channels: Catalyzers of Endolysosomal Transport and Function. Front. Pharm..

[179] Nguyen O.N., Grimm C., Schneider L.S., Chao Y.K., Atzberger C., Bartel K., Watermann A., Ulrich M., Mayr D., Wahl-Schott C. (2017). Two-Pore Channel Function Is Crucial for the Migration of Invasive Cancer Cells. Cancer Res..

[180] Garcia-Rua V., Feijoo-Bandin S., Rodriguez-Penas D., Mosquera-Leal A., Abu-Assi E., Beiras A., Maria Seoane L., Lear P., Parrington J., Portoles M. (2016). Endolysosomal two-pore channels regulate autophagy in cardiomyocytes. J. Physiol..

[181] Qureshi O.S., Paramasivam A., Yu J.C., Murrell-Lagnado R.D. (2007). Regulation of P2X4 receptors by lysosomal targeting, glycan protection and exocytosis. J. Cell Sci..

[182] Suurvali J., Boudinot P., Kanellopoulos J., Ruutel Boudinot S. (2017). P2X4: A fast and sensitive purinergic receptor. Biomed. J..

[183] Murrell-Lagnado R.D., Frick M. (2019). P2X4 and lysosome fusion. Curr. Opin. Pharmacol..

[184] Toyomitsu E., Tsuda M., Yamashita T., Tozaki-Saitoh H., Tanaka Y., Inoue K. (2012). CCL2 promotes P2X4 receptor trafficking to the cell surface of microglia. Purinergic Signal..

[185] Huang P., Zou Y., Zhong X.Z., Cao Q., Zhao K., Zhu M.X., Murrell-Lagnado R., Dong X.P. (2014). P2X4 forms functional ATP-activated cation channels on lysosomal membranes regulated by luminal pH. J. Biol. Chem..

[186] Gofman L., Cenna J.M., Potula R. (2014). P2X4 receptor regulates alcohol-induced responses in microglia. J. Neuroimmune Pharm..

[187] Moore M.N., Allen J.I., McVeigh A., Shaw J. (2006). Lysosomal and autophagic reactions as predictive indicators of environmental impact in aquatic animals. Autophagy.

[188] Meng Lin M., Kim H.H., Kim H., Muhammed M., Kyung Kim D. (2010). Iron oxide-based nanomagnets in nanomedicine: Fabrication and applications. Nano Rev..

[189] Zhu J., He K., Dai Z., Gong L., Zhou T., Liang H., Liu J. (2019). Self-Assembly of Luminescent Gold Nanoparticles with Sensitive pH-Stimulated Structure Transformation and Emission Response toward Lysosome Escape and Intracellular Imaging. Anal. Chem..

[190] Lee D.U., Park J.Y., Kwon S., Park J.Y., Kim Y.H., Khang D., Hong J.H. (2019). Apoptotic lysosomal proton sponge effect in tumor tissue by cationic gold nanorods. Nanoscale.

[191] Bhowmik T., Gomes A. (2016). NKCT1 (purified Naja kaouthia protein toxin) conjugated gold nanoparticles induced Akt/mTOR inactivation mediated autophagic and caspase 3 activated apoptotic cell death in leukemic cell. Toxicon.

[192] Totten J.D., Wongpinyochit T., Seib F.P. (2017). Silk nanoparticles: Proof of lysosomotropic anticancer drug delivery at single-cell resolution. J. Drug Target..

[193] Zhang X.F., Gurunathan S. (2016). Combination of salinomycin and silver nanoparticles enhances apoptosis and autophagy in human ovarian cancer cells: An effective anticancer therapy. Int. J. Nanomed..

[194] Martin-Banderas L., Holgado M.A., Duran-Lobato M., Infante J.J., Alvarez-Fuentes J., Fernandez-Arevalo M. (2016). Role of Nanotechnology for Enzyme Replacement Therapy in Lysosomal Diseases. A Focus on Gaucher’s Disease. Curr. Med. Chem..

[195] Muhlstein A., Gelperina S., Shipulo E., Maksimenko O., Kreuter J. (2014). Arylsulfatase A bound to poly(butyl cyanoacrylate) nanoparticles for enzyme replacement therapy—Physicochemical evaluation. Pharmazie.

[196] Tan Y.L., Ho H.K. (2018). Navigating albumin-based nanoparticles through various drug delivery routes. Drug Discov. Today.

[197] Elzoghby A.O., Samy W.M., Elgindy N.A. (2012). Albumin-based nanoparticles as potential controlled release drug delivery systems. J. Control. Release.

[198] An F.F., Zhang X.H. (2017). Strategies for Preparing Albumin-based Nanoparticles for Multifunctional Bioimaging and Drug Delivery. Theranostics.

[199] Tang W.L., Tang W.H., Li S.D. (2018). Cancer theranostic applications of lipid-based nanoparticles. Drug Discov. Today.

[200] Guven E. (2020). Lipid-based nanoparticles in the treatment of erectile dysfunction. Int J. Impot Res..

[201] Zhou Y., Fang X., Gong Y., Xiao A., Xie Y., Liu L., Cao Y. (2017). The Interactions between ZnO Nanoparticles (NPs) and alpha-Linolenic Acid (LNA) Complexed to BSA Did Not Influence the Toxicity of ZnO NPs on HepG2 Cells. Nanomaterials.

[202] Peynshaert K., Soenen S.J., Manshian B.B., Doak S.H., Braeckmans K., De Smedt S.C., Remaut K. (2017). Coating of Quantum Dots strongly defines their effect on lysosomal health and autophagy. Acta Biomater..

[203] Pankiv S., Clausen T.H., Lamark T., Brech A., Bruun J.A., Outzen H., Overvatn A., Bjorkoy G., Johansen T. (2007). p62/SQSTM1 binds directly to Atg8/LC3 to facilitate degradation of ubiquitinated protein aggregates by autophagy. J. Biol. Chem..

[204] Thurston T.L., Ryzhakov G., Bloor S., von Muhlinen N., Randow F. (2009). The TBK1 adaptor and autophagy receptor NDP52 restricts the proliferation of ubiquitin-coated bacteria. Nat. Immunol..

[205] Kim B.W., Hong S.B., Kim J.H., Kwon D.H., Song H.K. (2013). Structural basis for recognition of autophagic receptor NDP52 by the sugar receptor galectin-8. Nat. Commun..

[206] Wild P., Farhan H., McEwan D.G., Wagner S., Rogov V.V., Brady N.R., Richter B., Korac J., Waidmann O., Choudhary C. (2011). Phosphorylation of the autophagy receptor optineurin restricts Salmonella growth. Science.

[207] Zeng J., Shirihai O.S., Grinstaff M.W. (2019). Degradable Nanoparticles Restore Lysosomal pH and Autophagic Flux in Lipotoxic Pancreatic Beta Cells. Adv. Healthc. Mater..

[208] Jimeno-Romero A., Oron M., Cajaraville M.P., Soto M., Marigomez I. (2016). Nanoparticle size and combined toxicity of TiO2 and DSLS (surfactant) contribute to lysosomal responses in digestive cells of mussels exposed to TiO2 nanoparticles. Nanotoxicology.

